# Local and General Inflammatory Mediators Status in Patients with Oral Lichen Planus

**DOI:** 10.3390/biomedicines14040763

**Published:** 2026-03-27

**Authors:** Irena Duś-Ilnicka, Anna Rybińska, Jakub Wronowicz, Agnieszka Rusiecka, Piotr Donizy, Małgorzata Radwan-Oczko

**Affiliations:** 1 Division of General and Experimental Pathology, Department of Clinical and Experimental Pathology, Wroclaw Medical University, ul. T. Marcinkowskiego 1, 50-368 Wrocław, Poland; 2 Periodontology Department, Faculty of Dentistry, Wroclaw Medical University, ul. Krakowska 26, 50-425 Wrocław, Poland; 3 Center for Statistical Analysis, Biostatistics Teaching Team, Wroclaw Medical University, ul. K. Marcinkowskiego 1, 50-368 Wrocław, Polandagnieszka.rusiecka@umw.edu.pl (A.R.)

**Keywords:** oral cancer, oral lichen planus, saliva, interleukin-1 beta, cytokines, inflammation

## Abstract

**Background**: Oral Lichen Planus (OLP) is a chronic inflammatory, autoimmune disorder affecting the skin and mucosa classified within the broad group of Oral Potentially Malignant Disorders (OPMDs). Topical treatment is usually effective in resolving the oral inflammation associated with the process, and the possible relationship to a systemic immunological reaction has not been widely discussed. The aim of this study was to explore the relationship between local and systemic inflammatory signatures in OLP by identifying potential markers in salivary and serum samples, as well as the topical treatment used to relieve inflammation. **Methods**: The study design was a cross-sectional case–control hospital-based study. A total of 50 blood samples, comprising 31 patients with OLP (study group) and 19 individuals without OLP status (control group), were tested for HLA-B27 in this study. Salivary and serum levels of Prostaglandin E2 (PGE2), matrix metalloprotease 8 (MMP-8), and human IL-1 beta (IL-1β) were measured within and between the control and OLP groups. **Results**: Salivary IL-1β levels were significantly higher in the OLP group than in controls (*p* = 0.001; W = 101.5). Serum MMP-8 concentrations were significantly lower in patients with OLP. Serum PGE2 levels were elevated in the OLP group; however, the difference was only borderline statistically significant after correction. *HLA-B27* allele frequency in the study and control groups was compared with that in the Polish population. Using Fisher’s Exact Test for Count Data, *p*-value = 0.1404, no statistically significant differences were found between the control and study groups. **Conclusions**: These findings could suggest that inflammatory activity in OLP might be predominantly localized to the oral cavity rather than systemic. Elevated salivary IL-1β and reduced systemic MMP-8 levels could support the concept of local immune dysregulation; moreover, salivary IL-1β may serve as a potential non-invasive biomarker for the diagnosis and monitoring of OLP. Studies involving a larger number of subjects should be conducted to strengthen the provided conclusion.

## 1. Introduction

Oral Lichen Planus (OLP) is an immune-mediated chronic mucosal disorder classified under Oral Potentially Malignant Disorders (OPMDs). Diagnostics of OPMDs is based on clinical evaluation of oral mucosal features, which vary across oral pathologies, with pathomorphological analysis as the only unifying diagnostic method recognized as the gold standard [1,2]. OLP is present in all racial groups at an estimated incidence of 0.5–1.5% [3], with a higher incidence in perimenopausal women [4] and with some ethnic differences [5]. OLP is characterized by a diverse malignant transformation rate of 0–6.25%, as reported by different sources [6]. Clinical presentation of the disease shows white reticular changes [7], often mirrored with the possible representation of an erosive, more aggressive form. The shape of the lesion can vary widely, including reticular, papillary, erythematous, erosive, and ulcerative lesions, with corresponding symptoms [5]. The most common site is the oral mucosa, especially the buccal mucosa, but the gingiva, tongue, lip, and palate can also be involved. All discussed clinical representations reflect the possibility of periods of relapse and remission [8], and regular oral screening is necessary for disease control [9]. As such, secondary prevention is also limited to frequent regular visits to dental offices, which are ineffective in remote rural settings [10,11,12]. This results in individuals with limited economic means being excluded in societies where dental healthcare costs are not fully covered [13,14,15].

Salivary protein diagnostics have been the focus of attention for their potential as markers across diverse treatment approaches and diseases, including systemic applications [16]. Matrix metalloproteases (MMPs) are a large family of zinc-dependent endopeptidases that can degrade all components of the basement membrane and extracellular matrix [17]. Increased MMP expression influences the invasive activity of tumor cells, allowing them to penetrate surrounding organs and tissues and to affect growth, migration, apoptosis, and angiogenesis [17]. MMP-8 is a proinflammatory cytokine produced by polymorphonuclear leukocytes, involved in inflammatory regulation and tissue remodeling [18,19,20,21]. In the field of oral health, MMP-8 is involved in tissue repair of the mucous membrane in ulcerative lesions [17]. MMP-8 is also associated with granulation tissue formation and has been thoroughly discussed as a diagnostic marker in the field of extracellular matrix in diseased implants [22]. Although OLP has an immune-inflammatory background, only one manuscript has described the potential relationship between MMP-8 levels in OLP [23].

In turn, proinflammatory interleukin-1β (IL-1β) induces adhesion molecules that facilitate and amplify the inflammatory response [16]. In the field of oral health, there has been a suggestion of a strong relationship between the severity of adult periodontitis and the increasing gingival crevicular fluid levels of IL-1β [16]. IL-1β has been identified as a potential biomarker for oral squamous cell carcinoma (OSCC), as it is released by cultured OSCC cells and detected in the saliva of patients with OSCC [16]. It has been discussed as a possible marker of diagnostic and/or prognostic significance in this type of oral cancer [16], and shows potential as an indicator of both precancerous and cancerous lesions in the oral cavity [24,25]. It was suggested that IL-1β concentrations could exhibit significant spikes relative to pre- and post-operative values, potentially serving as markers of oral squamous cell carcinoma (OSCC) [16]. Although IL-1β plays a role in inflammatory responses, cell death, apoptosis, and bone resorption [26], it has scarcely been analyzed in the saliva of patients with OLP. Lastly, among the cytokines of importance investigated in the saliva of patients with OPMD, Prostaglandin E (PGE2) salivary levels are higher in patients with type 2 diabetes mellitus (type 2 DM) than in non-diabetic individuals with periodontal inflammation [27], as there is a correlation between diabetes mellitus and OLP [28], which has also been reported by us in the previous study [29].

The first-line treatment for changes in the oral mucosa, following oral cavity sanitation and the withdrawal of risk factors, include topical steroids, topical calcineurin inhibitors [30], or plant-derived suppositories such as flaxseed extracts and *aloe vera* gels [30,31], among others. The topical treatments used by the patient, in the context of various oral pathologies, affect oral homeostasis by influencing the oral microbiota, saliva production, whole-body fluid balance, and immunoglobulin production in saliva or blood [32,33].

The aim of this study is to explore the relationship between local and systemic inflammatory signatures in OLP by identifying potential markers in salivary and serum samples, in an exploratory manner, in relation to the local treatment of patients.

## 2. Methods and Samples

### 2.1. Clinical Evaluation

The study was conducted at the Oral Pathology Outpatient Clinic, a one-day clinic within the Oral Pathology Department, where patients were primarily recruited for various pathologies. The research was designed as a cross-sectional case–control hospital-based study. Because the clinic’s patients presented with diverse stages of oral pathology or general disorders with oral manifestations, healthy subjects in the control group were recruited who had no oral changes and maintained a high level of oral hygiene, as previously described in the first manuscript [29]. From this study group, 31 patients with OLP lesions and 19 healthy individuals without any oral mucosal pathology and with proper oral hygiene were included. A specialist in oral mucosal pathologies performed clinical investigations. A general medical history was collected focusing on the type of disease, the duration of OLP, and the presence of lichen planus lesions in other locations (skin or genitalia), as well as on dermatological treatments for generalized lichen planus based on the anamnesis [29]. All medical visits to the Oral Pathology Outpatient Clinic took place between 9 a.m. and 3 p.m. From the OLP and control groups of patients, both blood and saliva samples were collected in accordance with the previously applied protocol [34]. The OLP diagnosis was provided by an experienced oral pathologist based on the clinical presentation of the oral mucosa and accompanying symptoms, along with thorough dental diagnostic work including periodontal analysis. Patients were also classified as having the erosive form of OLP if such findings were observed on the day of examination. After the period of observation and withdrawal of any external factors that might influence oral health status, usually taking around 2 weeks, a biopsy for pathomorphological analysis was performed in cases requiring gold-standard diagnostics.

### 2.2. Saliva and Blood Collection

Blood samples were collected (using a closed vacuum system) into a vacuum tube containing EDTA anticoagulant for whole blood, and into clot activator vacuum tubes for serum. For each study participant, an identification number was assigned and used to sign the blood and saliva test tubes to ensure pseudonymity. The specimens being processed to yield serum were allowed to completely clot for 15–30 min by leaving them undisturbed at room temperature, and then centrifuged at 10,000× *g* for 10 min at 4 °C. The collected saliva samples were centrifuged under the same conditions. The obtained supernatants were aliquoted into three sterile 1.5 mL tubes and stored at −80 °C until use. Whole blood collected into a tube containing an EDTA anticoagulant was aliquoted into three 1.5 mL sterile tubes and stored at −80 °C until use, without prior centrifugation.

### 2.3. Presence of the HLA-B27 Gene

A total of 50 blood samples, comprising 31 patients with OLP (study group) and 19 individuals without OLP status (control group), were tested for HLA-B27 in this study. In the first step, genomic DNA was prepared using the DNeasy Blood & Tissue Kit (QIAGEN, Germantown, MD, USA) according to the manufacturer’s instructions. A UV-Vis spectrophotometer was used to determine DNA purity and concentration. After DNA extraction, a ready-to-use HLA Ready Gene GLA-B27 kit (Inno-train Diagnostic GmbH, Kronberg im Taunus, Germany) was used to determine the HLA-B*27 gene by polymerase chain reaction with sequence-specific priming (PCR-SSP). The molecular diagnostic system mentioned used the polymerase chain reaction (PCR), which can amplify specific DNA sequences. DNA concentration was adjusted to the manufacturer’s recommended concentration for the Ready Gene kit. The HLA Ready Gene kit consisted of PCR strips with pre-aliquoted, dried, and colored reaction mixes containing allele-specific primers and internal control primers; PCR buffer, PCR strip caps, molecular weight markers, worksheets, Taq polymerase, and instructions for use were also provided. The reaction was performed according to the manufacturer’s recommendations using a C1000 Touch Thermal Cycler (BIORAD, Hercules, CA, USA). The PCR products were subjected to electrophoresis in a PowerPac Basic apparatus (Bio-Rad) on a 2% agarose gel stained with Midori Green Advance DNA Stain (NIPPON Genetics EUROPE, Düren, Germany) at 200 V for 20 min. Visualization and archiving of the electrophoresis result were performed on the Gel Doc EZ Imager by Biorad. The size of the PCR product was determined using the molecular weight markers, specifically the 100 bp DNA Ladder included with the kit.

### 2.4. Prostaglandin E2 (PGE2) Saliva and Serum Level Diagnostics

For the quantitative determination of Prostaglandin E2 in the saliva and serum samples, a commercially available kit, the Parameter Prostaglandin E2 Immunoassay (R&D Systems, Minneapolis, MN, USA), was used. The kit was based on a competitive enzyme-linked immunosorbent assay (ELISA); in this competitive binding assay, the PGE2 present in the sample competed with horseradish peroxidase (HRP)-labeled PGE2 for a limited number of binding sites on a mouse monoclonal antibody.

A standard curve was generated by plotting the absorbance for each standard (2500 pg/mL, 1250 pg/mL, 625 pg/mL, 313 pg/mL, 156 pg/mL, 78 pg/mL, 39 pg/mL) on a linear y-axis against the concentration on a logarithmic x-axis. The concentration of PGE2 in the samples was calculated from the standard curve absorbance and multiplied by the dilution factor. The assay range was 39–2500 pg/mL with a sensitivity of 41.4 pg/mL.

### 2.5. Total MMP-8 Saliva and Serum Level Diagnostics

For the quantitative determination of human active and pro-matrix metalloproteinase 8 (total MMP-8) concentrations in the saliva and serum samples, a commercially available kit, the Quantikine^®^ Human Total MMP-8 Immunoassay (R&D Systems, Minneapolis, MN, USA), was used, which was based on a sandwich enzyme-linked immunosorbent assay (ELISA). In this technique, a monoclonal antibody specific to human MMP-8 was used, pre-coated onto a microplate.

The standards and samples were pipetted into the wells, and the immobilized antibody bound to MMP-8. After washing away any unbound substances, an enzyme-linked monoclonal antibody specific to human MMP-8 was added to the wells.

Then, the wells were washed to remove any unbound antibody–enzyme reagent. Afterward, a substrate solution was added to the wells, and the color developed in proportion to the amount of MMP-8 bound in the initial step. Subsequently, the color development was stopped, and the color intensity was measured using an Epoch Microplate Spectrophotometer (US BioTek Laboratories, Shoreline, WA, USA). The color intensity was proportional to the total MMP-8 concentration in the samples. A standard curve was generated by plotting the absorbance for each standard (10 ng/mL, 5 ng/mL, 2.5 ng/mL, 1.25 ng/mL, 0.625 ng/mL, 0.313 ng/mL, 0.156 ng/mL) on a linear *y*-axis against the concentration on a logarithmic *x*-axis. The concentration of total MMP-8 was calculated from the standard curve absorbance and multiplied by the dilution factor. The assay range was 0.156–10 ng/mL with a sensitivity of 0.013 ng/mL.

### 2.6. Human IL-1 Beta Saliva and Serum Level Diagnostics

For the quantitative determination of interleukin-1β (IL-1β) concentrations in saliva and serum samples, a commercially available kit, the Quantikine™ HS Human IL-1β/IL-1F2 Immunoassay (R&D Systems, USA), was used, based on a quantitative sandwich enzyme-linked immunosorbent assay (ELISA). In this technique, a microplate is pre-coated with a monoclonal antibody specific for human IL-1β.

A standard curve was generated by plotting the absorbance for each standard (8.0 pg/mL, 4.0 pg/mL, 2.0 pg/mL, 1.0 pg/mL, 0.50 pg/mL, 0.25 pg/mL, 0.125 pg/mL) on a linear *y*-axis against the concentration on a logarithmic *x*-axis. The concentration of IL-1β was calculated from the mean absorbance of the standard curve. The assay range was 0.1–8 pg/mL with a sensitivity of 0.063 pg/mL.

### 2.7. Saliva Analysis of IL-1β, PGE2, and MMP-8 Between OLP and Control Groups

Additional analysis was performed to assess potential correlations among IL-1β, PGE2, and MMP-8 in the control and OLP groups; however, this analysis used a new set of control group saliva samples. The analysis involved evaluating saliva from the control and OLP groups, and the control group was carefully selected. The methods used (ELISA) were performed as described in the previous subchapters.

### 2.8. Statistical Methods

Correlations between saliva and serum parameters were assessed using the Spearman correlation, as the data were non-normally distributed. Additionally, a monotonicity check was performed to ensure the validity of the Spearman correlation. Comparisons between the OLP and control groups were performed using the Wilcoxon sum rank test for independent samples. To evaluate whether OLP severity influenced biomarker concentrations, an additional subgroup comparison was conducted using the Kruskal–Wallis test, as the variables did not meet the assumptions of normality and the groups were independent. This analysis compared the saliva and serum levels of IL-1β, MMP-8, and PGE2 across three groups: erosive OLP, non-erosive OLP, and healthy controls. A corrected *p*-value < 0.05 was considered statistically significant.

### 2.9. Ethical Consideration

Each participant was able to ask questions regarding the survey, as required by the Declaration of Helsinki. Agreement from the bioethics committee at Wroclaw Medical University, no. KB760/2021, was received, and all patients provided written consent before participating in the study.

## 3. Results

The characteristics of the study group are presented in Table 1. This specific cohort was previously evaluated in the manuscript published in Dental and Medical Problems [29], though the diagnostic methods, the approach, and the aim were selected specifically for the purpose of this study.

### 3.1. HLA-B*27 Gene Considerations in the OLP Group

Sampling for the OLP group was random, consistent with the distribution in the Polish population. HLA-B*27 allele frequency in the study and control groups was compared with that in the Polish population. Using Fisher’s Exact Test for Count Data, *p*-value = 0.1404, no statistically significant differences were found between the control and study groups, indicating that the groups were homogeneous.

### 3.2. Sample Size Analysis

The effect size and test power for all Wilcoxon sum rank tests and Spearman correlations were calculated (for Spearman correlations, the equivalent sample size is the correlation coefficient). The report presented a sample size and power analysis based on a Monte Carlo simulation comparing the control and OLP groups. It evaluates Prostaglandin E2 (PGE2), total MMP-8, and human IL-1β biomarkers measured in the serum and saliva.

For the effect sizes:

- PGE2 in serum: an effect size of 0.310, which was evaluated as moderate;
- PGE2 in saliva: an effect size of 0.189, which was evaluated as small;
- MMP-8 in serum: an effect size of 0.490, which was evaluated as moderate;
- MMP-8 in saliva: an effect size of 0.262, which was evaluated as small;
- IL-1β in saliva: an effect size of 0.521, which was described as large.

Post hoc Monte Carlo power analysis showed adequate power for MMP-8 measured in serum (0.97) and IL-1β measured in saliva (0.98). Limited power was evaluated for the representation of PGE2 levels in serum (0.60), MMP-8 in saliva (0.45), and PGE2 in saliva (0.26). Moderate-to-large effects in the study were sufficient to support conclusions about IL-1β in saliva and MMP-8 in serum. Particularly, in this field, the statistical power was reported to be excellent. For PGE2 in saliva and MMP-8 in saliva, in the case of a lack of significance, it should not necessarily mean a lack of a biological difference.

### 3.3. Monotonicity Test—Prostaglandin E2 (PGE2), Total MMP-8, and Human IL-1β

The monotonicity/normality check was used to determine the applicability of the statistical tests.

**Figure 1:**
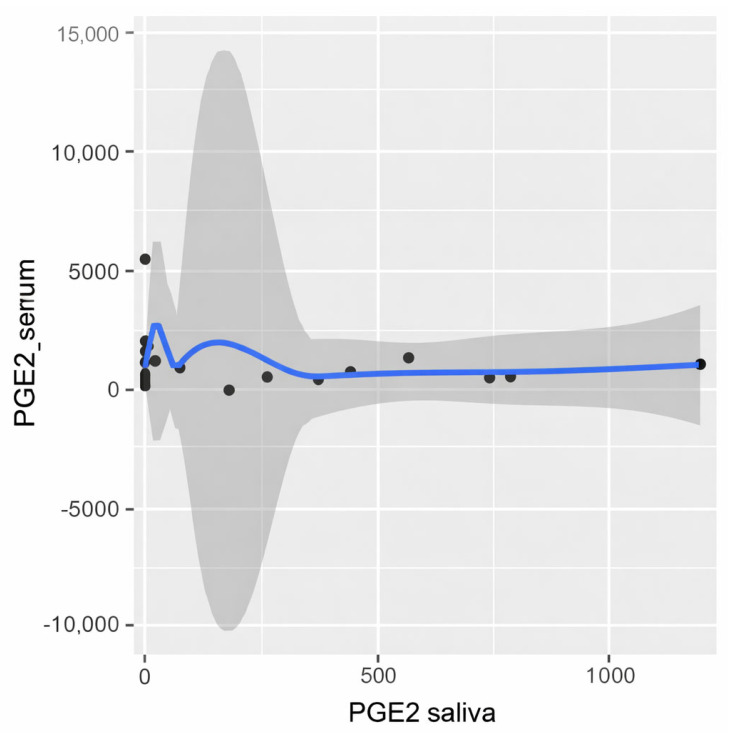
PGE2 scatter plot summarizing results from serum and saliva within OLP group.

**Figure 2:**
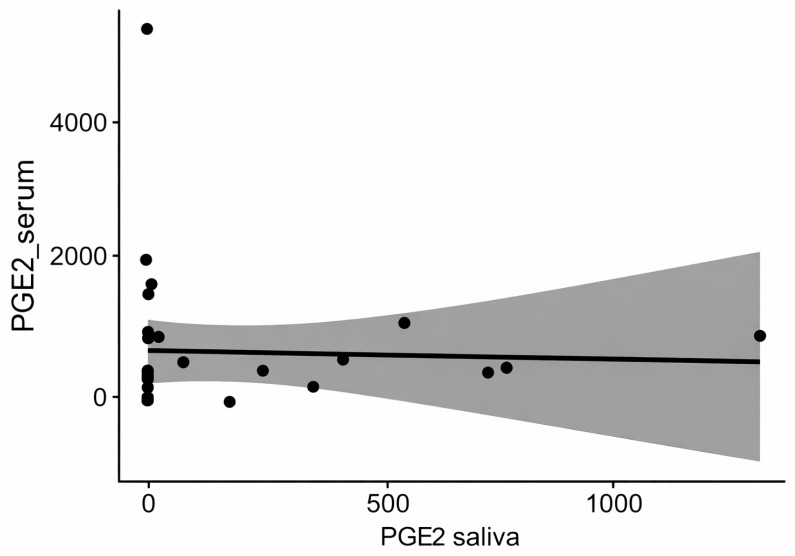
PGE2 correlation between serum and saliva within OLP group.

The distributions of PGE2 concentrations in both saliva and serum differed significantly from normal (*p* = 5.8 × 10^−8^, W = 0.59; *p* = 5.1 × 10^−8^, W = 0.59, respectively), and Spearman’s correlation was performed. The monotonicity of the relationship was assessed using a scatter plot (Figure 1). The result is shown in Figure 2.

**Figure 3:**
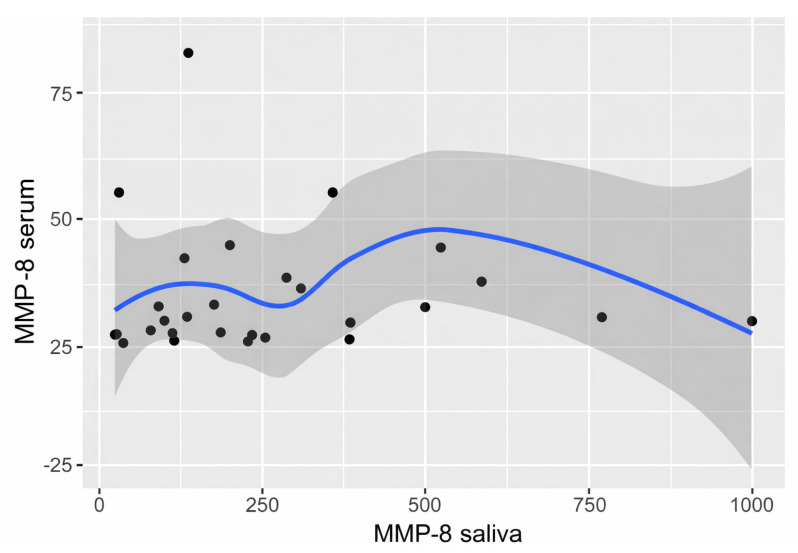
MMP-8 scatter plot summarizing results from the serum and saliva within OLP group.

**Figure 4:**
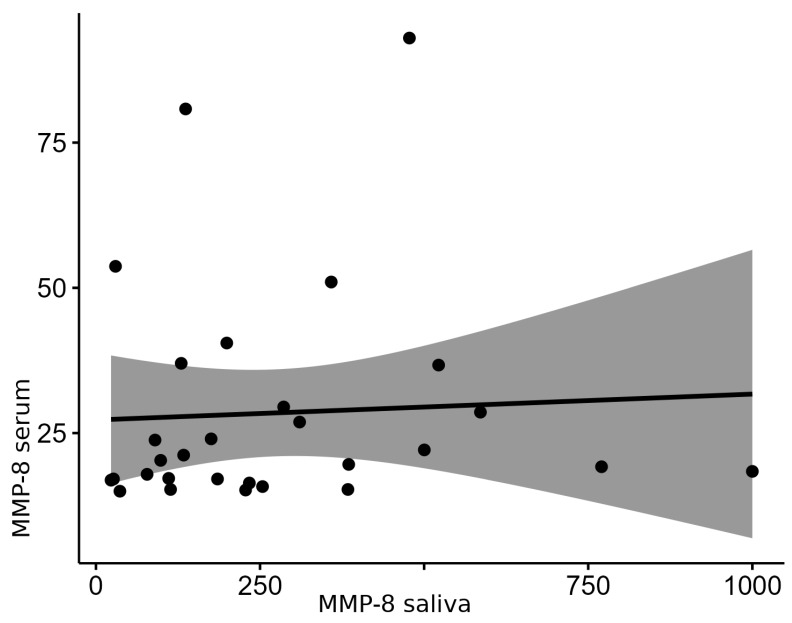
Correlation of MMP-8 levels between the serum and saliva within OLP group.

MMP-8 in both saliva and serum showed distributions that differed significantly from normal (*p* = 1.7 × 10^−4^, W = 0.87; *p* = 1.8 × 10^−6^, W = 0.70, respectively), so Spearman’s correlation was performed. The monotonicity of the relationship was tested and presented in a scatter plot (Figure 3). The relationship was roughly monotonic, as seen in Figure 4.

A comparison of the IL-1β in serum and saliva was not performed due to the limited range of results; it cannot be concluded whether there are statistically significant differences between saliva and blood.

### 3.4. Saliva and Serum PGE2 and MMP-8 Levels Comparison

As presented in Table 2, there were no statistically significant correlations between the saliva and serum levels of PGE2 or MMP-8 within the OLP patient group.

Because all variables exhibited non-normal distributions, Spearman’s rank correlation was used to analyze the relationships between the saliva and serum levels of PGE2, MMP-8, and IL-1β. No statistically significant correlations were observed for PGE2 (ρ = 0.26, *p* = 0.17) or MMP-8 (ρ = 0.22, *p* = 0.26) within the OLP group, suggesting no monotonic association between local and systemic concentrations of these mediators.

### 3.5. Serum PGE2 and MMP-8 Levels Comparison Between the OLP and Control Groups

**Figure 5:**
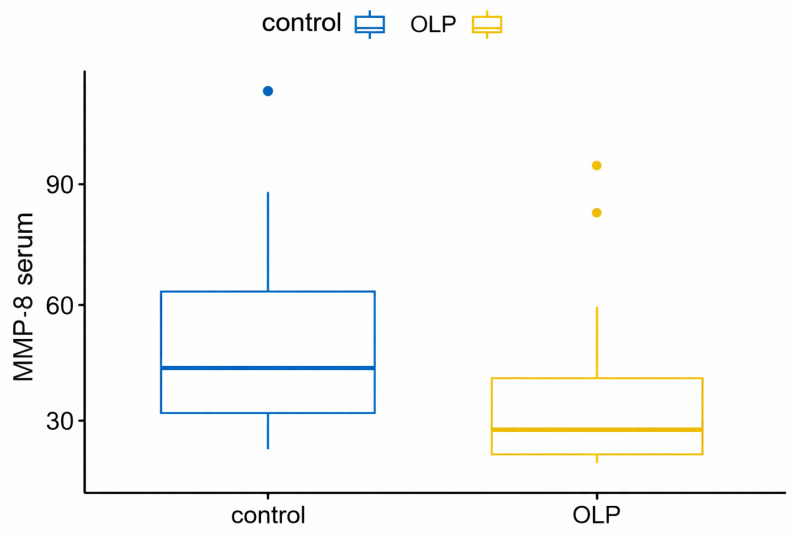
MMP-8 correlation in serum between OLP and control groups.

**Figure 6:**
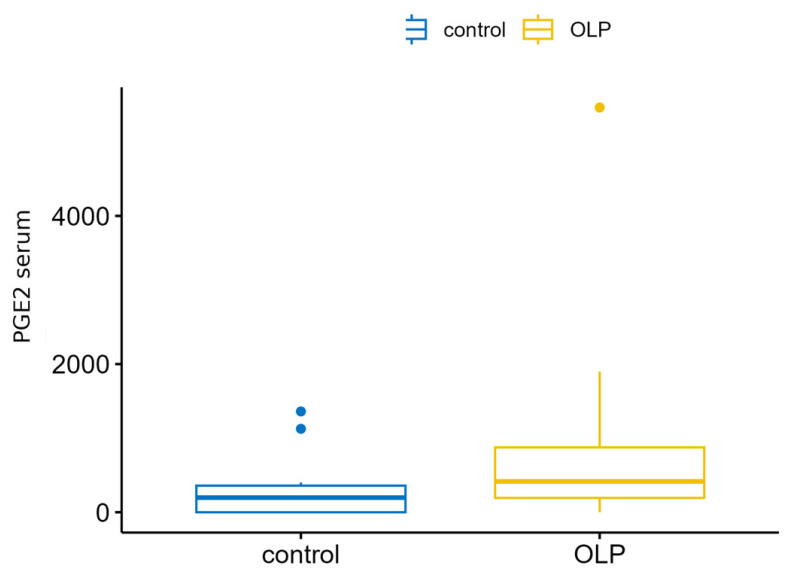
PGE2 correlation in serum between OLP and control groups.

Comparisons between the study and control groups’ serum levels of the analyzed immunoglobulins were performed using a *t*-test (if the assumptions were met) or the Wilcoxon sum rank test. The variables exhibited non-normal distributions; therefore, the Wilcoxon sum rank test was used. For the serum MMP-8 variable, MMP-8 levels were significantly lower in patients with OLP than in the controls (Figure 5). However, for the serum PGE2 variable (Figure 6), which was higher in the OLP group, the adjusted result was borderline significant (Table 3 and Table 4).

Additional analysis was performed using the Kruskal–Wallis test. Because nine patients were diagnosed with the erosive form of OLP, a further assessment was performed to determine whether serum or saliva parameters (MMP8, IL-1β, and PGE2) were significantly associated with this stage of the disorder. The results showed a statistically significant difference in serum MMP-8, with lower levels in patients with erosive OLP than in the control group (higher in the control group), as revealed by post hoc analysis. These parameters were in concordance with the results for the total OLP group.

### 3.6. Saliva IL-1β, MMP-8, and PGE2 Comparison Between OLP and Control Groups

Additional analysis was performed to assess the potential correlations among IL-1β, PGE2, and MMP-8 in the control and OLP groups. In this analysis, a new set of control-group saliva samples was provided. The median age of the patients was 42 years in the control group, with a median (Q1–Q3): 42 (35–53) and mean ± SD: 43.74 ± 12.21.

The variables showed a non-normal distribution. Comparisons between the study and control groups’ saliva levels of the analyzed immunoglobulins were performed using Spearman’s rank correlation, as presented in Table 5.

For the saliva IL-1β variable, there was a significant difference (*p*-value = 0.001, W = 101.5) with higher values in the OLP group when compared to control.

While salivary PGE2, MMP-8, and IL-1β showed significant correlations in both groups, only IL-1β showed a statistically significant difference between OLP patients and the controls after multiple testing correction, as shown in Table 5 and Table 6.

### 3.7. Investigated Characteristics of Topical OLP Treatments Used Against OLP

In the medical history of OLP treatment, the most commonly used corticosteroid has been 1 g of ointment containing 0.5 mg of betamethasone as betamethasone dipropionate. Topical calcineurin (Tacrolimus 0.1%) ointment was previously prescribed to seven volunteers (22.58%). Additionally, 15 volunteers reported using the local herbal remedy (flaxseed derivatives) or other moisturizing measures as suggested by the oral pathologist to relieve symptoms and feelings of keratinized, rough, dry mucosa (48.39%). Apart from those declared in Table 7, upon admission, patients used topical local treatment of oral mucosa of other remedies that were available over the counter (OTC), like topical vitamin A in capsules with vitamin E; oral probiotics; oral gels with chlorhexidine; oral gels with herbal extract of Baikal skullcap (Latin Scutellaria baicalensis); oral gels with hyaluronic acid; a commercial oral solution with active substances of cartilage extract, silver/copper nanocolloids, and carboxymethylcellulose; and ozonated olive oil. In the routine care described here, treatment was adjusted according to clinical response; therefore, it was not ethically justifiable to delay or withhold appropriate treatment solely to allow time for stratification and extended observation of the selected treatment. At this point in our research, the local treatment was simply observational and comprised the patients’ history descriptions.

## 4. Discussion

The International Agency for Research on Cancer reported that OLP is a chronic inflammatory disorder of unknown origin that manifests as white reticular lesions, potentially accompanied by atrophic, erosive, or ulcerative plaque-type areas, which are frequently observed symmetrically [35]. This oral disorder poses a diagnostic challenge, as, when considered in isolation, neither the clinical picture nor the histopathology reveals the full extent of the disease. The diversity of OLP representations—from reticular to erosive—might also overlap with clinical representation of other OPMDs for the untrained pathologist’s eye. Desquamative gingivitis, although not a separate disease entity, is rather a clinical manifestation of OLP [36,37]. These specific OLP manifestations have been described in our previous research based on the same study cohort [29]. Treatment of OLP is a lengthy process and, without the possibility of a complete cure, raises concerns about its cost-effectiveness. So far, the most widely used and practical treatment approach is topical therapy of the oral mucosa. The treatment prescribed is always based on the clinical stage of the lesions—white or red lesions with erosions or end ulcerations—and on the presence of various signs and symptoms reported by patients. In long-term observations, with proper care and patient cooperation, herbal remedies have commonly been used in treatment when lesion progression is present; meanwhile, in the longer term, corticosteroids or topical calcineurin inhibitors [3,10], under continuous monitoring, have been useful. In the OLP group presented here, this exact line of treatment has been provided: 15 volunteers reported using a local herbal remedy (linseed oil or other natural moisturizing preparations or ointments), which the oral pathologist had suggested to alleviate dryness of keratinized mucosa (48.39%). It was an alternative to OTC drugs and saliva substitutes prepared at home by patients from linseed (*Linum usitatissimum* L.), a plant rich in mucilage. The use of linseed mucus to alleviate xerostomia symptoms is a method patients have used for years, even without medical consultation [31]. The mechanism of its effectiveness remains incompletely understood [31], but it has recently been described in our previous article [31]. During clinical examination, 41.94% of OLP patients had been using topical corticosteroids while 22.58% had been using topical tacrolimus, primarily to reduce active, inflammatory–erosive disease. The rationale for using this type of drug is that OLP is a cell-mediated, chronic inflammatory disorder, and corticosteroids exhibit both anti-T-cell-mediated and anti-inflammatory immune effects. With adequate treatment by both the dentist and the patient, accelerated healing of OLP can be expected [28]. Although the patients may use these remedies, local inflammation has been observed, as evidenced by elevated Il-1β concentrations.

A genetic biomarker in the HLA class I family, HLA-B27 (Human Leukocyte Antigen B27), as analyzed in this work, is associated with an increased risk of developing certain autoimmune and inflammatory disorders—particularly those associated with spondyloarthritis [32] and ankylosing spondylitis [33], among others. No significant deviations from the Polish population have been observed, suggesting that the inclusion of the randomized group in this research was appropriate for the HLA-B27 biomarker analysis.

IL-1β, as a pleiotropic key proinflammatory cytokine, promotes the synthesis of other inflammatory cytokines. To date, it has been analyzed in the saliva of diverse patient groups to detect early-stage oral squamous cell carcinoma (OSCC). Although OLP has been classified as an OPMD with one of the lowest progression rates, salivary analysis comparing OLP and control groups has indicated that IL-1β shows significant differences in spike counts between the groups. In the presented manuscript, statistical differences in IL-1β levels between the OLP and control groups have been observed. These results are consistent with those reported by Kamatani T. et al. [23], showing that IL-1β is one of the few biomarkers that can be used not only in OSCC but also in OPMDs, including OLP, with lower progression rates. Both Il-1β and PGE2 levels have previously been analyzed in saliva during initial orthodontic treatment across age groups [38]. PGE2 is a proinflammatory and immunoregulatory cytokine derivative of the arachidonic acid cascade that increases chemotactic properties and vascular permeability by vasodilation [27,38]. The analysis in this manuscript assessed potential correlations between saliva and serum PGE2 levels in patients with OLP, which is considered locally confined, but may also cause general symptoms. In this study, serum PGE2 levels did not differ significantly between groups. The analysis has shown limited statistical significance, suggesting that a larger sample size is needed to yield more robust results. As IL-1β is discussed as a potential diagnostic biomarker in OPMDs, it is important to note that saliva is a biomaterial that requires careful handling during sampling, which may strictly affect diagnostic outcomes [39]. To facilitate sampling, patients should not eat or drink for 2 h beforehand, flush the oral cavity with lukewarm water, and have the diagnostic procedure provide aliquoting.

In previous studies, MMP-8 was reported to be higher in patients with rheumatoid arthritis with chronic periodontitis than in healthy controls without chronic periodontitis [40]. Additionally, in the field directly related to the OPMD, there are reports on the potential clinical application of collagen-degradation markers, such as MMP-8, in patients with OLP [23]. However, these parameters show excellent potential for clinical use in OPMD; to date, they have been analyzed primarily in periodontal disease [41,42]. As discussed in this study, serum MMP-8 concentrations had been significantly lower in patients with OLP than in healthy controls. In contrast, salivary MMP-8 did not differ significantly between groups. These findings suggest that MMP-8 involvement in OLP might be predominantly local rather than a systemic event, supporting the concept of compartment-specific inflammatory regulation in this disease [43]. The study by Gorbatova et al. also suggested that oral MMP-8 concentration can play an essential role in evaluating OLP severity [17]. The authors noted that this concentration was higher in more severe clinical stages of OLP and decreased significantly after treatment, which was associated with reduced inflammation in OLP lesions. In our study, serum MMP-8 levels had been significantly lower in both the overall OLP group and the erosive OLP group than in the control group. This may be because the OLP-related inflammation in our patients was low, though the patients had been under continuous treatment and regular recall visits. Moreover, all potential local (irritating or worsening) traumatic factors, such as amalgam fillings, sharp elements of prosthetic restorations, sites of dental plaque accumulation, and dietary irritants, had been eliminated beforehand and could still be controlled. The IL-1β results in the present study suggest a localized event of OLP, consistent with the previously described analysis of MMP-8 and PGE2 [44].

Importantly, because we included patients with erosive OLP in our study, the results from this group further support this conclusion; that is, despite the presence of this more clinically active subtype, MMP-8 levels did not differ from those in the control group, indicating that the disease severity had not translated into increased systemic MMP-8 levels. This may reflect an effective inflammatory control resulting from continuous treatment and regular follow-ups. All parameters presented here (PGE2, IL-1β, MMP-8) were previously analyzed primarily in patients’ saliva, with limited analysis in serum, a strength of this manuscript.

The main limitations of this study concern two aspects: first, the study included a relatively small group of patients with OLP; and second, the clinical characteristics of the lesions may have influenced the obtained results, particularly the levels of salivary biomarkers. In the majority of patients investigated, the OLP lesions had been predominantly white and relatively asymptomatic, without evident inflammation, erosions, or ulcerations.

## 5. Conclusions

The statistical differences between the OLP and control groups in IL-1β levels suggest that IL-1β may serve as a biomarker for OLP diagnosis and as an inflammatory biomarker. Statistical significance in serum MMP-8 levels, with lower levels in the OLP erosive patients compared with the control group, is also consistent with the results for the total OLP group, suggesting a possible localized event of OLP rather than a systemic disorder. The discrepancy supports a compartmentalized inflammatory response, in which local MMP-8 production does not translate into systemic elevation.

The borderline statistical behavior of serum PGE2 likely reflects the small sample size, limited variability, and non-normal distribution of the measurements. Therefore, while serum PGE2 showed a trend towards higher values in OLP patients, the results should be interpreted with caution. The high prevalence of local ointment use in the OLP group suggests the need for localized remedies for patients with OLP, including economic, safe, and herbal options.

## Figures and Tables

**Table 1:** Characteristics of OLP patients.

Patients Characteristics	Number of OLP Patients-31
**Age in years**	Mean 62.39 ± 11.02; Range (37–82)
**Biological sex:**	Women-28; Man-3
**Smoking:**	No-30; Yes-1
**Clinical OLP forms:**	White (striae, plaque) n = 22; Erosive n = 9

**Table 2:** Analysis of saliva and serum concentrations of PGE2 and MMP8 in OLP group.

Variable 1	Variable 2	ρ	Statistic	*p*-Value	Method	*p*-Value Adjusted
**PGE2 in saliva**	PGE2 in serum	0.26	3326.80	0.17	Spearman’s rank correlation	0.33
**MMP-8 in saliva**	MMP-8 in serum	0.22	3176.78	0.26	Spearman’s rank correlation	0.51

**Table 3:** Analysis of the saliva and serum concentrations of PGE2 and MMP-8 between the groups.

Group	Variable	*p* Value	Statistic
Control	PGE2 serum	0.0001	0.72
OLP	PGE2 serum	5.05015 × 10^−8^	0.59
Control	MMP-8 serum	0.0055	0.85
OLP	MMP-8 serum	1.82038 × 10^−6^	0.70

**Table 4:** Wilcoxon sum rank test for analysis of serum concentrations of PGE2 and MMP8 between study and control groups.

	Statistic	*p*-Value	Method	*p*-Value Adjusted
**PGE2 serum**	180	0.0311	Wilcoxon sum rank test with continuity correction	0.06210
**MMP-8 serum**	452	0.0006		0.00127

**Table 5:** Analysis of the saliva concentrations of IL-1β, PGE2, and MMP-8 between the OLP and control groups.

Variable Analyzed in Saliva	Variable 2	Statistic	*p*-Value	Method	*p*-Value Adjusted
PGE2	Group: OLP and Control	345.5	0.19004	Wilcoxon sum rank test with continuity correction	0.570119
MMP-8		189.5	0.071454		0.214362
IL-1β		101.5	0.000372		0.001117

**Table 6:** Analysis of the saliva concentrations of IL-1β, PGE2, and MMP-8 within the OLP and control groups.

Group	Variable Analyzed in Saliva	*p*-Value	Statistic
Control	PGE2	2 × 10^−5^	0.661437
OLP		5.85 × 10^−8^	0.592065
Control	MMP-8	0.001204	0.801555
OLP		0.001655	0.866033
Control	IL-1β	0.003169	0.829507
OLP		0.004188	0.880826

**Table 7:** Description of the medical treatment used to lower the immunologic response to the OLP.

Topical Treatment Used For Oral Lichen Planus	
**Linseed (in oil and infusion)**	
No	16 (51.61%)
Yes	15 (48.39%)
**Topical corticosteroids**	
No	13 (41.94%)
Yes	18 (58.06%)
**Topical tacrolimus**	
No	24 (77.42%)
Yes	7 (22.58%)

## Data Availability

The original contributions presented in this study are included in the article. Further inquiries can be directed to the corresponding author.
